# Validation of the Oncomine^™^ focus panel for next-generation sequencing of clinical tumour samples

**DOI:** 10.1007/s00428-018-2411-4

**Published:** 2018-08-13

**Authors:** Hannah L. Williams, Kathy Walsh, Austin Diamond, Anca Oniscu, Zandra C. Deans

**Affiliations:** 1 UKNEQAS for Molecular Genetics, Royal Infirmary of Edinburgh, Edinburgh, UK; 2 NHS Lothian, Department of Molecular Pathology, Royal Infirmary of Edinburgh, Edinburgh, UK; 3 University of St Andrews, School of Medicine, North Haugh, St Andrews, Fife, KY16 9TF UK; 4 NHS Lothian, Molecular Genetics, Western General Hospital, Edinburgh, UK

**Keywords:** Next-generation sequencing, Molecular pathology, Clinical validation, FFPE

## Abstract

The clinical utility of next-generation sequencing (NGS) for a diverse range of targets is expanding, increasing the need for multiplexed analysis of both DNA and RNA. However, translation into daily use requires a rigorous and comprehensive validation strategy. The aim of this clinical validation was to assess the performance of the Ion Torrent Personal Genome Machine (IonPGM^™^) and validate the Oncomine^™^ Focus DNA and RNA Fusion panels for clinical application in solid tumour testing of formalin-fixed, paraffin-embedded (FFPE) tissue. Using a mixture of routine FFPE and reference material across a variety of tissue and specimen types, we sequenced 86 and 31 samples on the Oncomine^™^ Focus DNA and RNA Fusion assays, respectively. This validation considered a number of parameters including the clinical robustness of the bioinformatics pipeline for variant detection and interpretation. The Oncomine^™^ Focus DNA assay had a sample and variant-based sensitivity of 99.1 and 97.1%, respectively, and an assay specificity of 100%. The Oncomine^™^ Focus Fusion panel had a good sensitivity and specificity based upon the samples assessed, however requires further validation to confirm findings due to limited sample numbers. We observed a good sequencing performance based upon amplicon, gene (hotspot variants within gene) and sample specific analysis with 92% of clinical samples obtaining an average amplicon coverage above 500X. Detection of some indels was challenging for the routine IonReporter^™^ workflow; however, the addition of NextGENe® software improved indel identification demonstrating the importance of both bench and bioinformatic validation. With an increasing number of clinically actionable targets requiring a variety of methodologies, NGS provides a cost-effective and time-saving methodology to assess multiple targets across different modalities. We suggest the use of multiple analysis software to ensure identification of clinically applicable variants.

**Electronic supplementary material:**

The online version of this article (10.1007/s00428-018-2411-4) contains supplementary material, which is available to authorized users.

## Introduction

Personalised medicine for the treatment of cancer provides directed therapy for patients based upon the genetic and epigenetic alterations of their disease. This requires laboratories to provide rapid assessment of the molecular landscape of the tumour to enable informed treatment decisions. Many of the current clinical testing algorithms are laborious, with multiple tests performed separately for a single patient. Next-generation sequencing (NGS) enables the testing of multiple genes, from multiple patient samples across dual modalities in one assay. The Ion Torrent NGS system is compatible with FFPE tissue which is currently routine processing of pathology specimens [1] and requires minimal nucleic acid input (10 ng total of DNA or RNA) which is beneficial for testing frequently very small, diagnostic samples.

With increasing affordability enabling the implementation of NGS into clinical laboratories, validation of both assay and bioinformatic analytical pipelines are the primary challenges clinical laboratories face. To comply with ISO15189 accreditation [2], validation of the detection of somatic variants must, as a minimum, assess limit of detection, analytical sensitivity and specificity, repeatability and reproducibility and set appropriate thresholds and quality control parameters for reliable analysis of clinical specimens. Validation must also include the handling of large amounts of data produced by multi-gene panels [3].

This study presents the validation of the Oncomine^™^ Focus DNA and RNA panel on the Ion Torrent Personal Genome Machine (IonPGM^™^, Thermo Fisher Scientific) for joint implementation within the Department of Molecular Pathology and United Kingdom National External Quality Assessment Service (UK NEQAS) for Molecular Genetics, Royal Infirmary of Edinburgh, UK. The Oncomine^™^ Focus DNA and RNA assay comprises two separate panels (DNA and RNA) which were designed to interrogate hotspot mutations (35 genes), copy number variations (19 genes) and gene fusions in 23 genes. Combined, these two panels can identify current actionable genetic variants and potential future targets for personalised therapy.

## Materials and methods

### Sample selection

Seventy-eight anonymised FFPE tissues comprising of melanoma (*n* = 18), colorectal cancer (CRC) (*n* = 28), non-small-cell lung cancer (NSCLC) (*n* = 22) and gastrointestinal stromal tumours (GIST) (*n* = 10) were processed from a range of specimen types: resections (*n* = 57), biopsies (*n* = 13), cell blocks (*n* = 6), fine needle aspirate (FNA) (*n* = 1) and polyps (*n* = 1). Neoplastic content was assessed and ranged from 20 to 90% as determined by a pathologist. In addition, nine reference samples were tested including four commercially available standards AcroMetrix^™^ Oncology Hotspot Control catalog no. 969056, AcroMetrix^™^ Frequency Ladder (six variant allele frequencies: 2.8, 5.4, 11, 18.4, 29.5 and 47.9%), Horizon Structural Multiplex Reference Standard catalog no. HD753 and Horizon EGFR Gene-Specific Multiplex Reference Standard catalog no. HD300 and six in-house reference standards: REF 2 (68 variants), REF 3 (6 variants), REF 4 (133 variants), REF 5 (131 variants), REF 6 (9 variants) and REF 7 (9 variants) (S1 file). The limit of detection was calculated using data from the AcroMetrix^™^ Hotspot Frequency Ladder. RNA assay specificity and sensitivity was assessed using clinical samples, four in-house reference standards and the ALK-RET-ROS1 Fusion FFPE RNA Reference standard (Horizon Diagnostics catalog no. HD784, RNA REF 1–4). The Human Brain Total RNA (Thermo Fisher Scientific, catalog no. AM7962) was used to assess RNA reproducibility.

### Nucleic acid extraction and quantification

DNA was extracted from melanoma, CRC and GIST samples using QIAmp DNA FFPE Tissue Kit (Qiagen) following manufacturer’s protocol (excluding de-paraffinisation). Dual DNA and RNA isolation was performed from NSCLC tissues using the RecoverAll Total Nucleic Acid Isolation Kit for FFPE (Thermo Fisher Scientific). DNA and RNA concentrations were determined by fluorometric quantitation using Qubit 2.0 Fluorimeter with Qubit DNA dsDNA BR Assay Kit and Qubit RNA BR Assay Kit (Qiagen) as appropriate.

### Next-generation sequencing

Complementary DNA (cDNA) synthesis prior to library preparation for RNA panel was carried out using SuperScript^™^ VILO^™^ cDNA Synthesis Kit (Thermo Fisher Scientific, 11754050). Library preparation was carried out using the Oncomine Assay^™^ (comprising the DNA Oncomine^™^ Focus Assay (Thermo Fisher Scientific) and RNA Oncomine^™^ Fusions assay (Thermo Fisher Scientific)) following manufacturer’s instructions using a total of 10 ng input DNA and or RNA per sample (minimum 0.83 ng/μl sample DNA concentration). A maximum of seven DNA samples were prepared per run (six samples if both DNA and RNA analyses were required) on an Ion 318^™^ v2 chip (Thermo Fisher Scientific, catalog no. 4488150). The DNA panel can identify hotspot mutations in the following genes: *AKT1*, *ALK*, *AR*, *BRAF*, *CDK4*, *CTNNB1*, *DDR2*, *EGFR*, *ERBB2*, *ERBB3*, *ERBB4*, *ESR1*, *FGFR2*, *FGFR3*, *GNA11*, *GNAQ*, *HRAS*, *IDH1*, *IDH2*, *JAK1*, *JAK2*, *JAK3*, *KIT*, *KRAS*, *MAP2K1*, *MAP2K2*, *MET*, *MTOR*, *NRAS*, *PDGFRA*, *PIK3CA*, *RAF1*, *RET*, *ROS1* and *SMO*; however, not all genes were assessed for the purposes of this validation. The RNA panel can identify rearrangements in *ALK*, *RET*, *ROS1*, *NTRK1*, *NTRK2*, *NTRK3*, *FGFR1*, *FGFR2*, *FGFR3*, *MET*, *BRAF*, *RAF1*, *ERG*, *ETV1*, *ETV4*, *ETV5*, *ABL1*, *AKT3*, *AXL*, *EGFR*, *ERBB2*, *PDGFRA* and *PPARG*, not all fusions were assessed for this validation. Nineteen copy number variant (CNV) targets are also included in the Oncomine^™^ Focus Panel; however, these were not validated in this study. Template preparation was performed on the Ion Chef System (Thermo Fisher Scientific) using the Ion PGM Hi-Q Chef Kit and/or the Ion One Touch^™^ 2 System using the Ion PGM Template OT2 200 Kit. Sequencing was performed using the Ion PGM Hi-Q Sequencing Kit on the Ion Torrent Personal Genome Machine (Ion PGM).

### Data analysis

Analysis was carried out using Ion Torrent Suite^™^ Browser version 5.0 and Ion Reporter^™^ version 5.0. The Torrent Suite^™^ Browser was used to perform initial quality control including chip loading density, median read length and number of mapped reads. The Coverage Analysis plugin was applied to all data and used to assess amplicon coverage for regions of interest. Variants were identified by Ion Reporter filter chain 5% Oncomine^™^ Variants (5.0)*.* A cut off of 500X coverage was applied to all analyses. All identified variants were checked for correct nomenclature using Alamut Visual v.2.7.1 (Interactive Biosoftware). Any discrepancies in variant identification, between Ion Reporter and Alamut, were validated manually using the Integrative Genomics Viewer [4, 5] and NextGENe® v2.4.2 (SoftGenetics®). For the purposes of this validation, amplicons covering clinically actionable regions with known mutation status (termed target amplicons; Table 1) were assessed as a subset of all amplicons (amplicons which target hot spot variants, i.e. SNVs and indels) covered in the Oncomine^™^ Focus hot spot BED file.

**Table 1:**
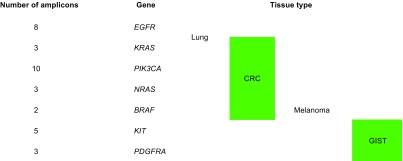
Target amplicons

Table details ‘target amplicons’ per tissue type and number of amplicons covering each gene of interest based upon current clinical and EQA requirements within UKNEQAS and Molecular Pathology at the Royal Infirmary of Edinburgh

## Results

### Oncomine^™^ focus DNA panel

#### Sequencing performance

Of the 78 FFPE samples, for 4 samples (exclusively NSCLC), no amplicons were covered to 500X (minimum criteria for this validation) and were considered failed samples. All failed samples had an input DNA concentration below 2.34 ng/μl; however, not all samples below this DNA input failed sequencing. No relationship between DNA concentration and amplicon coverage was identified.

**Fig. 1:**
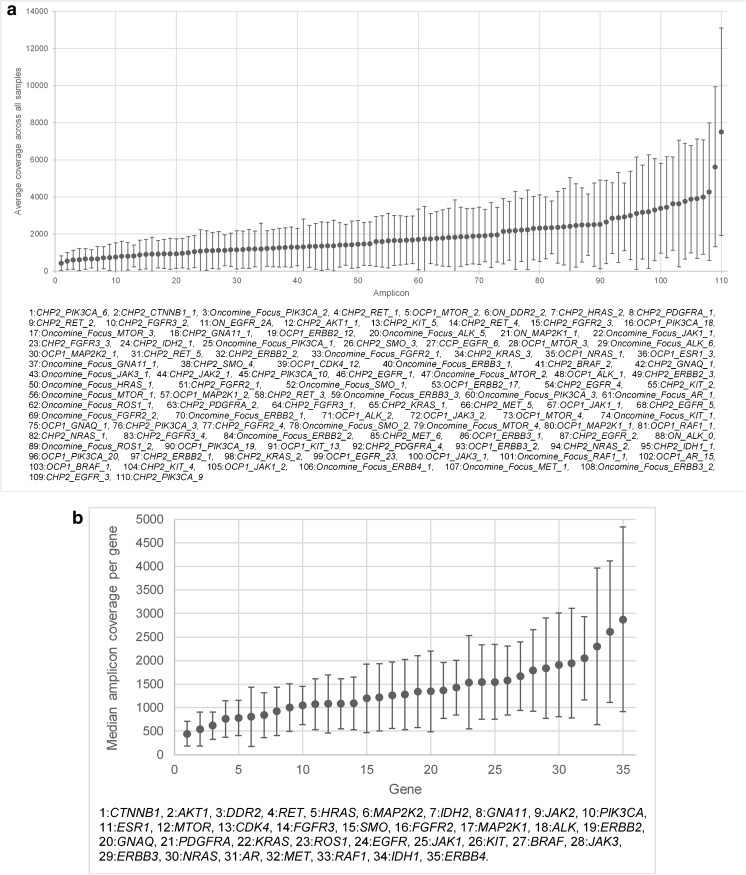
Assessment of amplicon- and gene-based sequencing performance by average amplicon coverage. **a** Average amplicon coverage across all clinical samples tested (n78). Ninety-nine percent of amplicons were covered on average to a minimum of 500X ^1^Average amplicon coverage was assessed for all hotspot amplicons in the Oncomine^™^ Focus assay. **b** Median amplicon coverage across all genes. Median coverage per gene (n35) comprising of a number of hotspot variants across exons per gene. A high variability in amplicon coverage was observed within and between genes. Intra-gene variability is depicted by interquartile range

The overall panel performance was assessed by average amplicon (n110) coverage across all cases (n78). The majority (99%) of all amplicons were covered on average to a minimum of 500X. The *PIK3CA* amplicon, CHP2_PIK3CA_6, which covers nucleotides in the later portion of exon 8 was the only amplicon with an average coverage below 500X across all cases (Fig. 1a). A high variability in amplicon coverage within and between gene variants (n35) was observed across the combined study cohort. For example, intra-gene variation in *EGFR* amplicon coverage across eight amplicons ranged from *Median (Md)* 686–4853, inter-gene variation in *PIK3CA* exon 8 (*Md* 327) and *KIT* exon 11 (*Md* 4008) (Fig. 1b). A trend was observed between median amplicon coverage and amplicon length (Spearman’s rho; *p* = 0.072) and between mean amplicon length and amplicon GC content (Spearman’s rho; *p* = 0.071).

**Fig. 2:**
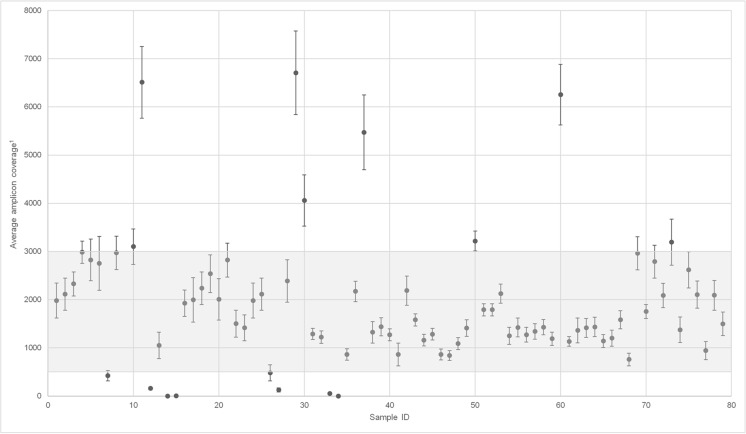
Sample-based sequencing performance. Sample-based sequencing performance was assessed by the average amplicon coverage (all hotspot amplicons) for all genes (n35) for each sample. A large proportion of samples had an average amplicon coverage for all genes between 500 and 3000X. Seven samples exceeded 3000X coverage with a maximum 6707X coverage. Eight samples had average amplicon coverage below 500X with two samples failing to sequence any genes

The average amplicon coverage per sample was also assessed; 89.7% (70/78) of all samples had an average amplicon coverage above 500X. A large proportion of samples (62/78, 79.4%) had an average amplicon coverage for the Oncomine Focus assay between 500 and 3000X (Fig. 2). No significant association between DNA concentration, sample type or tissue type could be identified in the seven samples exceeding an average amplicon coverage of 3000X.

Sample performance using the Focus panel was assessed based upon the proportion of amplicons reaching a minimum of 500X coverage for all amplicons and target amplicons, respectively. All amplicons were covered to a minimum of 500X in 12.8% of samples, and all had an input DNA concentration above the recommended 10-ng total input (range 1.97–125.5 ng/μl). Sixty-four samples (82%) had a proportion of amplicons covered to a minimum of ×500 (range 4.5–99.1%, *Md* 90%). Of the target amplicons (Table 1), 19 (24.4%) samples had all target amplicons covered to a minimum of 500X, all cases of which had an input DNA concentration above the recommended 10-ng input (range 1.29–125 ng/μl). Seventy four (94.9%) had a proportion of target amplicons covered to a minimum of 500X (range 2.9–97.1%, *Md* 88.2%). Based upon mean sequencing performance metrics, there was no significant difference in average amplicon coverage, total mapped reads or percentage of amplicons at 500X between samples based upon tissue type or sample type (Table 2).

**Table 2:** Sequencing performance metrics

	Total mapped reads (CV)	Average amplicon coverage depth (CV)	Percent of all amplicons ≥ ×500	Percent of target amplicons ≥ ×500
Total (n78)				
Tissue type				
Lung (n22)	390,884 (0.89)	1,545 (0.83)	61	64.3
GIST (n10)	507,137 (0.35)	1,861 (0.35)	81	88.8
Melanoma (n18)	396,993 (0.32)	1,447 (0.33)	82.6	85.6
CRC (n28)	441,153 (0.72)	1,613 (0.73)	73.7	81.7
*p* value	0.120	0.175	0.186	0.033^a^
Specimen type				
Cell block (n6)	469,270 (1.06)	2,166 (0.8)	61.2	62.8
Biopsy (n13)	374,194 (0.79)	1,371 (0.8)	59	69.2
Resection (n57)	430,195 (0.59)	1,569 (0.59)	77.3	81.9
*p* value	0.840	0.613	0.664	0.692

Sequencing performance metrics (total mapped reads, average amplicon coverage, percentage of all amplicons ≥ 500X, percentage of target amplicons ≥ 500X). Metrics are presented by tissue type and specimen type. Mean values are reported. A significant difference in % target amplicons at 500X between tissue type was identified; however, adjustment for false discovery rate (FDR) using Bonferoni correction deemed this not significant (Kruskal-Wallis *p* = 0.033; *n78*). There was however a trend in NSCLC samples having a lower percentage of amplicons at ≥ 500X than the other tissue types

^a^*p* value significant at 0.05. For specimen type analysis 2 samples, one fine needle aspirate and one polyp were excluded from statistical analysis due to limited numbers of samples of this type. Coefficient of variation stated for total mapped reads and average amplicon coverage

Tissue-specific sequencing performance was assessed by the percentage of target amplicons (specific to tissue type; Table 1) with minimum 500X depth of coverage per sample; 35 samples (44.8%) had all target amplicons covered to minimum 500X. GIST and melanoma samples had a greater proportion of samples achieving minimum 500X for all target amplicons (80 and 61.1%, respectively). Melanoma and GIST had the highest average percentage of tissue-specific target amplicons at 500X (per sample) (91.3 and 90.3%, respectively) whilst CRC and NSCLC had 78.8 and 68.4%, respectively.

#### Limits of detection

The limit of detection (LOD) were ascertained by repeated sequencing of the AcroMetrix^™^ Frequency Ladder which was analysed at the three lowest expected allele frequencies (EAFs) (2.8, 5.4 and 11%) for the presence of variants across all three repeats (S2 file).

Of the target genes assessed (Table 1), all (7) *EGFR* exon 21 variants failed to be detected at any of the three frequencies. These variants were reliably identified at 18.4% EAF; however, the observed variant allele frequency (VAF) was much lower than expected (average observed VAF 6.6%). Due to the pertinence of *EGFR* mutations in NSCLC, this was deemed an unsatisfactory LOD and required further investigation. Using the Horizon Discovery *EGFR* Gene specific multiplex reference standard (HD300) containing exon 21 variants, c.2582 T > A and c.2573 T > G at an EAF of 5%, all five variants (in addition to those above, c.2369C > T, c.2236_2250del and c.2155G > A) were detected at VAF between 4.5 and 6.2%, confirming a minimum LOD of 5% for the *EGFR* gene for these variants. LODs for *BRAF*, *EGFR*, *KRAS* and *NRAS* were 5.6% across all exons assessed and 11% for *PDGFRA* whilst *KIT* and *PIK3CA* demonstrated varying LODs depending upon exon assessed (Table 3).

**Table 3:**
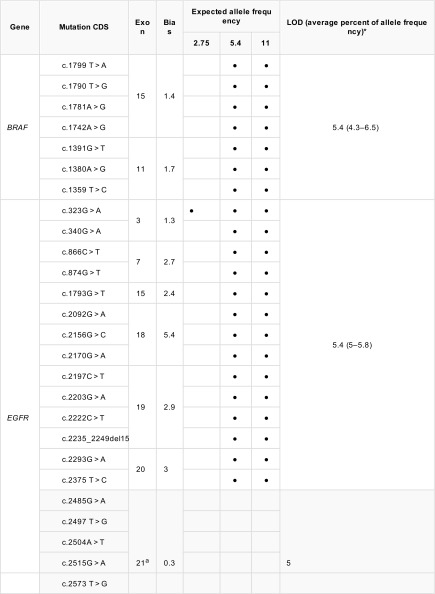

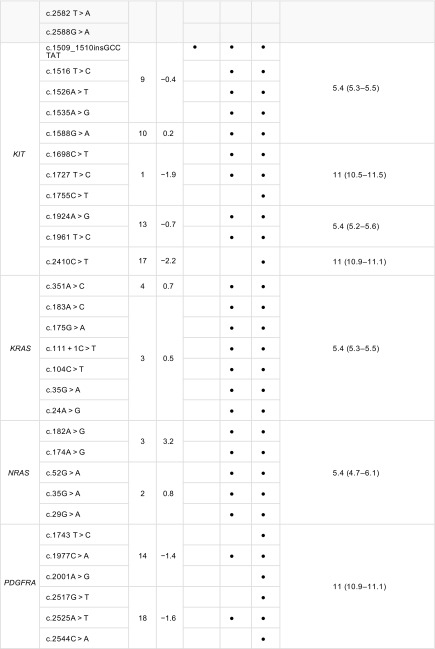

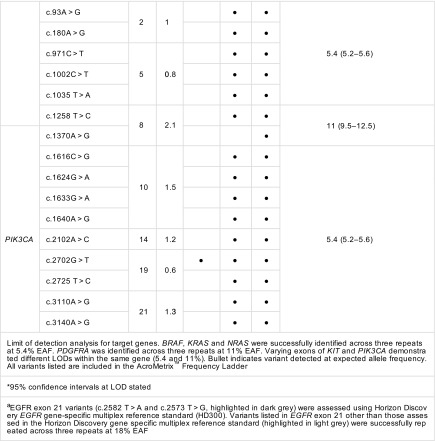
Limits of detection determination for target genes

Limit of detection analysis for target genes. *BRAF*, *KRAS* and *NRAS* were successfully identified across three repeats at 5.4% EAF. *PDGFRA* was identified across three repeats at 11% EAF. Varying exons of *KIT* and *PIK3CA* demonstrated different LODs within the same gene (5.4 and 11%). Bullet indicates variant detected at expected allele frequency. All variants listed are included in the AcroMetrix^™^ Frequency Ladder

*95% confidence intervals at LOD stated

^a^EGFR exon 21 variants (c.2582 T > A and c.2573 T > G, highlighted in dark grey) were assessed using Horizon Discovery *EGFR* gene-specific multiplex reference standard (HD300). Variants listed in *EGFR* exon 21 other than those assessed in the Horizon Discovery gene specific multiplex reference standard (highlighted in light grey) were successfully repeated across three repeats at 18% EAF

Of the variants assessable in the AcroMetrix^™^ Frequency Ladder, all variants in only one gene (*GNA11*) could be detected at the 2.8% EAF for all variants. Ten genes (40% of total genes identified) had all variants detected across triplicates at 5.4% EAF (*ALK*, *BRAF*, *CTNNB1*, *EGFR*, *FGFR1*, *FGFR2*, *IDH1*, *KRAS*, *MET*, *NRAS* and *SMO*). In addition to these, a further eight genes (76% of total genes identified) had all variants detected across all three runs at 11% EAF (*AKT1*, *ERBB2*, *GNA11*, *HRAS*, *IDH2*, *JAK2*, *MAP2K1* and *PDGFRA)*. LODs for all genes included in the AcroMetrix^™^ Frequency Ladder are detailed in S2 file.

In addition, during LOD analysis, we identified observed allelic frequencies with an element of positive or negative bias across repeats; e.g. observed VAFs were consistently higher than expected in some genes (*BRAF*, *EGFR*, *KRAS*, *NRAS* and *PIK3CA*) *and* consistently lower than expected in others (*KIT* and *PDGFRA*).

#### Robustness

Library preparations and sequencing runs were performed four times using the AcroMetrix^™^ Oncology Hotspot Control, which contained 146 targeted variants across 25 genes (S3 table), to determine assay inter-run reproducibility. One-hundred and forty-three variants were detected across all runs.

Using Ion Reporter^™^ (IR^™^) routine workflow, three false negatives were identified (*FGFR3* c.1928A > G, *PDGFRA* c.1698_1712del15 and *IDH2* c.474A > G) being absent from 2, 2 and 1 repeat, respectively. Conferring a routine workflow reproducibility for the variants assessed of 97.9%. The FASTQ files from the four repeats were further analysed by NextGENe® software (SoftGenetics®). Using this analysis, the variants that comprised the initial three false negatives from the IR routine workflow were identified in all four repeats; however, three different false negatives were produced using this analysis (*MET* c.3757 T > G, *MET* c.3778G > T and *RET* c.1894_1906 > AGCT) being absent from 1, 1 and 4 repeats, respectively (S3 file).

Intra-run repeatability was assessed by duplicate analysis of the 5.4% EAF (11/25) and 11% EAF (20/25) Acro-Metrix Hotspot Frequency Ladder samples containing 25 genes (hotspot variants within gene) comprising 140 variants; 44% (*mean VAF 6.9%*, *CV 0.22)* and 80% (*mean VAF 11%*, *CV 0.22)* repeatability was observed for all genes (hot spot variants within gene) at EAF levels 5.4 and 11%. On a variant basis for duplicate analysis of the 5.4 and 11% EAF, a repeatability of 96/140 (68.5%, *mean VAF 6.6%*, *CV 0.22*) and 132/140 (94.2%, *mean VAF 10.6%*, *CV 0.23*), respectively, was identified (S4 file).

**Fig. 3:**
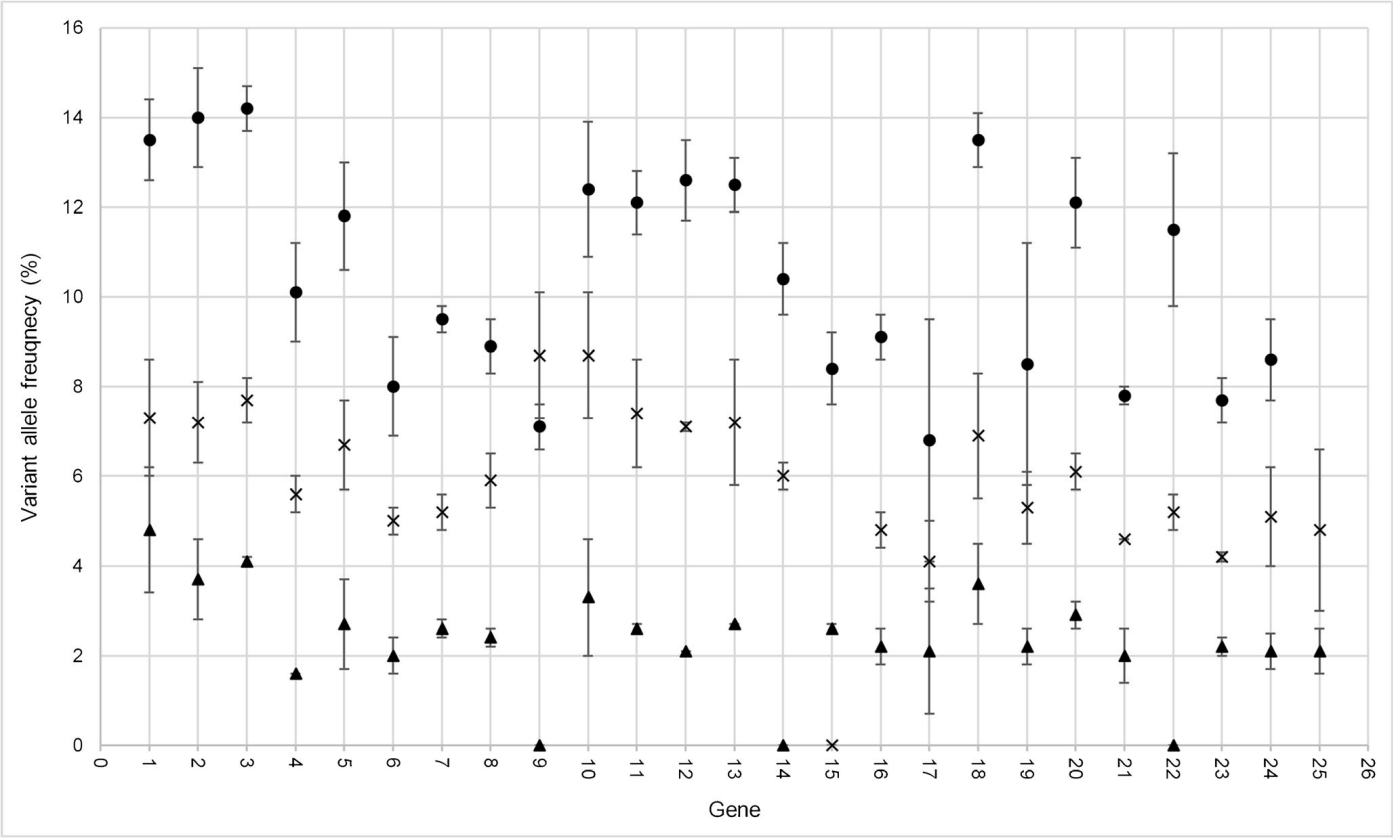
Inter-gene variation in expected allele frequency (EAF). Average variant allele frequency for 25 genes represented in the AcroMetrix^™^ hotspot frequency ladder. Standard deviation of represented variants within each gene is depicted. 1 *NRAS*, 2 *ALK*, 3 *IDH1*, 4 *CTNNB1*, 5 *PIK3CA*, 6 *FGFR3*, 7 *PDGFRA*, 8 *KIT*, 9 *APC*, 10 *EGFR*, 11 *MET*, 12 *SMO*, 13 *BRAF*, 14 *FGFR1*, 15 *JAK2*, 16 *GNAQ*, 17 *RET*, 18 *FGFR2*, 19 *HRAS*, 20 *KRAS*, 21 *AKT1*, 22 *MAP2K1*, 23 *IDH2*, 24 *ERBB2*, 25 *GNA11*. Black triangle 2.8% EAF, cross 5.4% EAF, black circle 11% EAF

**Fig. 4:**
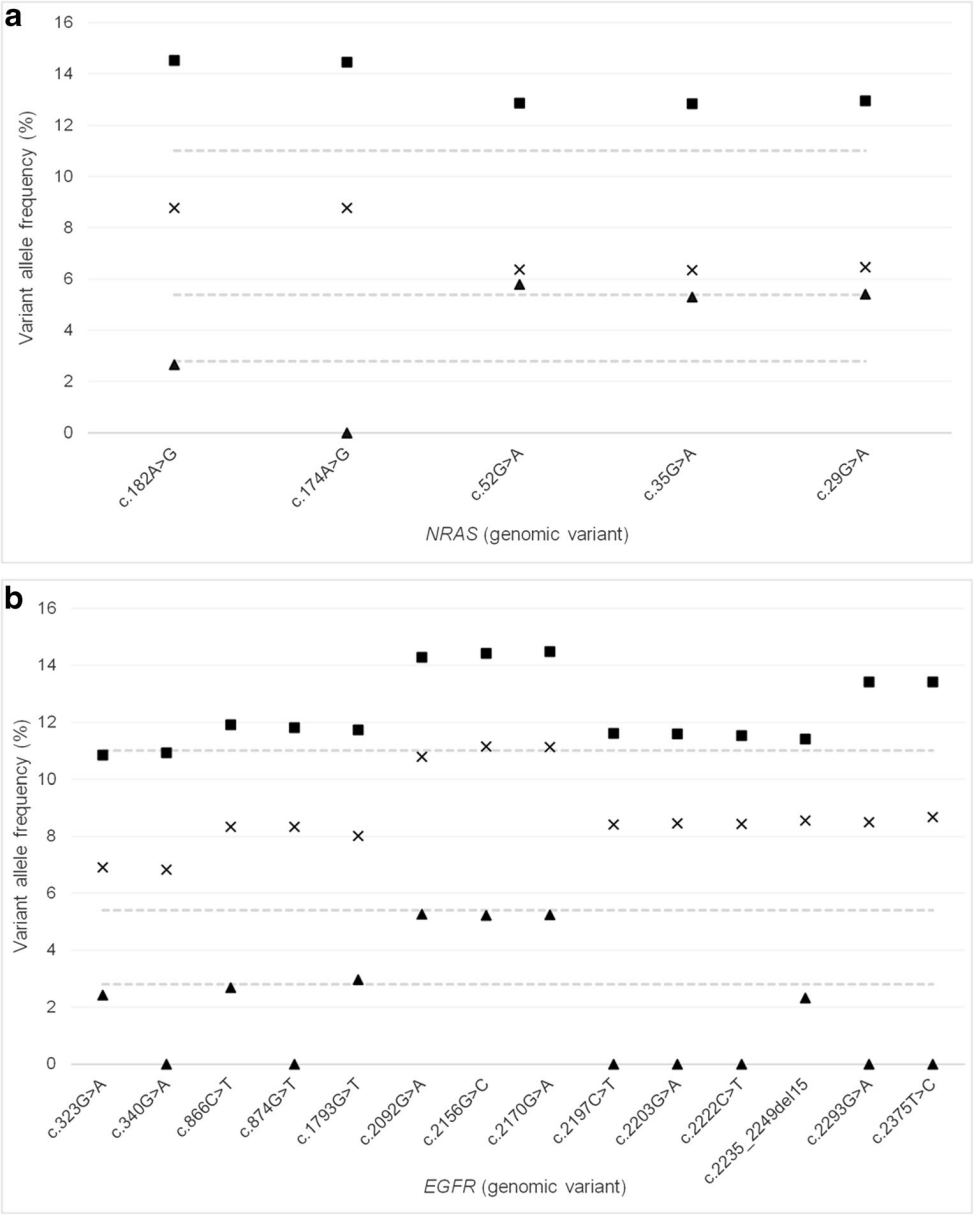
Intra-gene variation in expected allele frequency (EAF). **a** *NRAS* variants at 2.8, 5.4 and 11% EAF. **b** *EGFR* variants at 2.8, 5.4 and 11% EAF excluding exon 21 variants. A large variance is observed within genes at the 2.8% EAF; this variance decreases with increasing EAFs. A large proportion of genes demonstrate positive or negative bias from the EAF. Variation patterns observed between exons of same gene

For some variants, there was a large difference between the expected and observed VAFs demonstrating a positive or negative bias, thus preventing accurate VAFs to be derived. This was observed at both inter- and intra-gene level (Figs. 3 and 4).

#### Analytical sensitivity and specificity

Sensitivity assessment of the Oncomine^™^ Focus DNA Panel was determined at both variant (reference samples and clinical material) and sample level (clinical material only). For the purposes of sensitivity analysis, only pre-validated variants were assessed. Any additional identified variants not previously validated were not included in analysis. A total of 588 variants (across 86 samples) were sequenced, 6 of these failed due to quality and were removed from further analysis. At the variant level, 571 of 576 known variants were correctly identified conferring an analytical variant-based sensitivity of 99.13% (95% CI 99.1–99.15%). Five false negative results were identified (Table 4).

**Table 4:** False negative results for variant and sample-based sensitivity

False negatives	Sample type	Sample number	Gene	Expected variant (genomic nomenclature)
Variant sensitivity	Reference	REF1	*PDGFRA*	c.1698_1712del
	Reference	REF1	*IDH2*	c.474A > G
	Reference	REF 2	*FGFR3*	c.1928A > G
Variant and sample sensitivity	Clinical	6	*KIT*	c.1652_1663del
	Clinical	71	*KIT*	c.1676_1694delinsA

At the sample level, 71 out of 74 samples were successfully sequenced (69 gave concordant genotypes) giving an analytical sensitivity of 97.1% (95% CI 97.06–97.3%).

Variant-based assay specificity was assessed on 157 alternate variant negative genes (hot spot regions previously assessed) and conferred a 100% sensitivity with no false positives identified in hotspot regions assessed. Analytical assay specificity was determined based on the analysis of 77 FFPE samples; no false positives were detected conferring 100% analytical specificity. Overall, we identified a positive predictive value of 100% and a negative predictive value of 97.5%.

#### Bioinformatic performance

Of the false negatives identified in sensitivity analysis, sample 6 was previously validated using Sanger sequencing and contained a *KIT* c.1652_1663del p.Pro551_Val555delinsLeu mutation. This variant was absent in the initial output from Ion Reporter^™^; adjustment of analysis parameters and re-analysis resulted in successful identification of the variant, indicating that this mutation had been successfully sequenced but filtered out by the bioinformatics pipeline. The presence of the variant was confirmed by analysis of the FASTQ file with NextGENe® software (SoftGenetics®). Sample 71 that contained a second kit mutation c.1676_1694delinsA p.(Val559_565delinsGlu) was not identified by either the original Ion Reporter^™^ algorithm or the adapted pipeline. The presence of this variant was also identified by NextGENe® software (SoftGenetics®).

Due to a number of clinical targets residing within *KIT* and *PDGFRA* genes involving indels, we sequenced a further five cases with known indels. Using the normal Ion Reporter^™^ workflow for Oncomine DNA single sample analysis, 1/5 (20%) variants were identified. One additional variant was identified using a modified Ion Reporter^™^ workflow (soft-clipping parameters were decreased to enable greater sensitivity at ends of reads), three remained unidentified. The FASTQ files from these samples were analysed via NextGENe® software (SoftGenetics®), whereby 3/5 (60%) variants were successfully identified (Table 5). No additional false positives were identified using these workflows. Basic detection parameters, i.e. minimum SNV coverage and SNV allele frequency, were comparable between the two analysis software. No single workflow successfully identified all five variants.

**Table 5:** Challenging variant identification

Gene	Expected variant (genomic nomenclature)	IR^™^ normal workflow	IR^™^ deletion workflow	NextGENe®
*KIT*	c.1655_1660del		●	
	c.1728_1766dup			
	c.1726_1731dup	●	●	●
	c.1656_1676del			●
*PDGFRA*	c.2526_2537del			●

One of five variants was identified by IR^™^ routine workflow; an additional variant was identified by the IR^™^ deletion workflow. Three variants were identified by NextGENe® (SoftGenetics®) analysis, two of which had not previously been identified by either IR^™^ workflows

Six variants were identified with incorrect nomenclature by the Ion Report algorithm 5.0. Validation and correction of these calls was carried out using NextGENe® v2.4.2 (SoftGenetics®) and Alamut Visual v.2.7.1 (Interactive Biosoftware). A large proportion of nomenclature inconsistences were limited to deletions, insertions and duplications (Table 6).

**Table 6:** Nomenclature inconsistencies by Ion Reporter^™^

Gene	Expected variant	Ion Reporter^™^ variant
*KIT*	c.1672_1674dupAAG p.(Lys558dup)	c.1670_1671insGAA p.([Lys558dup)]
	c.1679_1681delTTG p.(Val560del)	c.1675_1677delGTT p.(Val559del)
	c.1735_1737 p.(Asp579del)	c.1733_1735delATG p.(Asp579del)
	c.1730_1738del p.(Pro577_Asp579del)	c.1728_1736del p.(Pro577_Asp579del)
*EGFR*	c.2303_2311dup p.(Ser768_Asp770dup)	c.2300_2301insCAGCGTGGA p.(Ala767_Ser768insSerValAsp)
*BRAF*	c.1798_1799delGTinsAA p.(Val600Lys)	c.1798_1799delGTinsAA p.(Val600Lys) plus c.1798G > A p.(Val600Met)

Five variants were identified by the IonReporter^™^ analysis workflow with the incorrect nomenclature and checked by Alamut Visual v2.7.1 (Interactive Biosoftware). A large proportion of nomenclature errors were deletions, insertions and duplications

## Oncomine^™^ RNA fusion panel

Thirty-one FFPE samples (6 biopsies, 8 cell blocks, 9 resections and 8 reference samples) previously validated by FISH were tested using the Oncomine^™^ Focus RNA fusions panel. A higher sequencing failure rate was observed with the RNA panel than the DNA; eight (25.8%) cases failed sequencing. These failures were not associated with age of sample. All failed samples had a DNA concentration below 8 ng/μl; however, not all samples below this concentration failed fusion analysis. Of the 23 samples which passed quality control, all fusion positive cases (*n* = 6) were correctly identified conferring an assay sensitivity of 100%. At the fusion level, all 31 fusions were identified across 23 samples conferring 100% specificity. One sample was identified as having an additional variant MET(8)–MET(9), which would not have been identified by current testing methodologies as this is not part of current clinical testing algorithms. Intra- and inter-run reproducibility was assessed using an EML4(6)–ALK(10) positive sample and demonstrated 100% concordance between and within runs. In addition, repeated analysis of the FirstChoice Human Brain Reference Total RNA showed 100% concordance between expression control presence, imbalance assay and fusion assay calls. Further confirmation of validation parameters is required prior to the clinical implementation of the RNA Fusion assay.

## Discussion

With an increasing requirement of clinical laboratories to perform multiple gene testing in both DNA and RNA, NGS panels designed for FFPE material provide a solution. Many commercially available NGS panels include > 400 targets, making the task of validating a solid tumour panel for implementation in a clinical setting very challenging. In this study, we validated the Oncomine^™^ Focus Panel for DNA and the Oncomine^™^ Fusion panel for RNA application using a diverse cohort of validation material consisting of both clinical and reference material enabling comprehensive validation of both sequencing platform and bioinformatics performance.

We validated the assay on both wet bench and bioinformatics processes across a broad spectrum of validation parameters including sequencing performance, analytical sensitivity and specificity, reproducibility, repeatability, robustness and limit of detection. A number of guidelines for the application of NGS sequencing and analysis clinical testing are available; however, a general consensus as to validation size, its application in the somatic setting and how parameters are assessed remains difficult to elucidate [6, 7, 11]. This validation was conducted using samples representative of the clinical samples routinely processed on site at the Royal Infirmary of Edinburgh including NSCLC, CRC, Melanoma and GIST. The main aim of the validation was to determine the overall applicability of the Ion PGM and Oncomine^™^ Focus Panel for DNA and Oncomine^™^ Fusion Panel for RNA to routine clinical testing. The appeal of the Oncomine^™^ Focus panel for clinical application is the requirement of a small starting input of DNA, which is particularly applicable when tissue availability is limited for example with NSCLC specimens.

Overall, we observed good amplicon coverage and sequencing performance for the Focus panel; however, sub-optimal performance for specific cases could not be associated with either sample or tissue type and may be derived from pre-processing procedures, prior to reaching the testing facility. Inadequate sample fixation and the type of fixative used have been shown to have an impact on DNA/RNA quality and the performance of downstream molecular procedures [12, 13]. For our FFPE sample cohort, we identified a clinically suitable analytical sample sensitivity and specificity of 100%, which is comparable with studies of similar panels and platforms [14, 15]. We did identify differences in the performance of variant identification between SNVs and indels, with the detection of indels presenting a greater challenge for routine bioinformatic workflows in comparison to SNV detection; this has been previously identified in a number of studies. In order to commence integration of NGS into routine clinical testing algorithms, parallel testing using both single-gene methods and NGS for prospective cases may be implemented to further cement the findings from this initial validation.

The applicability of the Oncomine^™^ Focus panel was easier to assess due to the wide range of clinical material available for validation. Two of the 78 samples assessed for this validation were below the minimum input requirements for the assay of which one sample failed to sequence any amplicons. The DNA quantification kit used in this study is known to lack precision below 5 ng/μl, which comprised 32 (41%) of the total samples assessed by the DNA panel. For future assessment of DNA quantification, the Qubit dsDNA HS Kit (Thermo Fisher Scientific), which has been shown to be sufficient in determining sample DNA concentration for NGS, would be a more sensitive method to better assess sequencing performance in relation to DNA input [8, 16]. To explore the clinical impact minimum DNA input requirements of the assay has on the number of cases which would be applicable to this method, an audit was carried out on 865 clinical samples for which DNA concentration had been quantified previously prior to testing using current routine methods. Ninety percent of cases would be considered applicable to sequencing by the Ion PGM platform with DNA concentrations greater than the 10 ng total requirement of the assay. Of those that would be below this threshold, 65% are derived from NSCLC samples.

We successfully sequenced 94.8% of samples in our FFPE validation cohort, which is comparable with other studies [10, 17]. A number of previous studies validating NGS platforms for solid tumour application have used 500X coverage as a minimum coverage criterion, which theoretically provides sufficient coverage to detect a 2% MAF, although coverage below this can be informative when variant alleles are at a higher frequency [3, 11, 18]. A broad range in average amplicon coverage was observed at both the inter- and intra-gene level. We hypothesise that amplicons with lower average amplicon coverage could be more affected by amplification-associated issues such as comprising highly repetitive sequences resulting in reduced PCR proficiency and quality for subsequent sequencing [11].

Non-small-cell lung cancer comprises a large proportion of the clinical workload within molecular pathology at the Royal Infirmary of Edinburgh, with the requirement of the assessment of multiple targets via a variety of methods supporting the application of multi-gene panels on NGS systems. From this validation however, NSCLC would appear to be the most challenging cases within the validation cohort with respect to meeting the initial demands of DNA input through to sequencing performance. Samples from this tissue type had the lowest percentage of all amplicons and target amplicons covered to a minimum coverage of 500X with the four samples failing to sequence any amplicons being exclusive to NSCLC. Studies involving assessment of NGS application for NSCLC have reported similar findings [9]. Despite that coverage below 500X can be informative when variant alleles are at a higher frequency, the findings from this validation demonstrate a higher degree of difficulty in the identification of lower-frequency variants in those amplicons which are below 500X coverage. For example using a minimum variant coverage of 10X in order for a variant call to be made by IonReporter^™^, coverage of 200X would be sufficient (assuming good quality reads) of detecting an alternate variant at approximately 5% frequency. The challenges presented by this sample group in terms of meeting input requirements and deriving quality sequencing data put pressure on meeting the clinical demands of returning timely results if repeat testing is required due to failed samples. Current methods for NSCLC testing (qPCR, pyrosequencing and FISH) enable results to be published to clinicians from the site of this validation within 5 days. To achieve a balance between cost per sample and time to results, turnaround times would be required to be increased from 5 to a minimum of 10 days. In order to meet testing demands, urgent requests will continue to be assessed using single-gene tests. Based upon the reduced sequencing performance of NSCLC in this validation, further validation to identify an accurate optimal threshold of sample quality prior to input will be required in order to triage samples more likely to fail and test these using single-gene test methods. NSCLC samples showed a high failure rate when assessed using the Fusions RNA panel which we hypothesise may be attributed to a higher proportion of larger samples in our validation cohort. In addition, this may also be as a result of sample processing methods prior to molecular testing such as length and extent of fixation of sample [19]. Obtaining clinically relevant ALK-positive material for validation is challenging; for example, out of 82 NSCLC cases tested for ALK rearrangements within Molecular Pathology in 2017, approximately 1% would test positive for ALK rearrangements. In addition to this, ROS testing is not currently carried out within Molecular Pathology making the identification of ROS positive cases for validation even more difficult. The limitations in the availability of clinical FFPE material to validate the panel further demonstrate the challenges in validating the RNA fusions panels for clinical application. In comparison to NGS fusion analysis, current methods for detecting ALK rearrangements in NSCLC, i.e. fluorescent in situ hybridisation (FISH), are relatively quick and cost-effective; based upon this, the RNA Fusion panel is not currently implementable as a clinical assay for assessment of ALK rearrangements.

In addition to the issues identified with sequencing performance of NSCLC specimens, we also identified issues in determining LODs for exon 21 of *EGFR* when using the AcroMetrix^™^ Oncology Hotspot Control*.* This exon is pertinent to NSCLC as it is required for the assessment of patient suitability for treatment with tyrosine kinase inhibitors such as erlotinib (Tarceva®). Detection of *EGFR* exon 21 variants using the AcroMetrix^™^ Hotspot Frequency Ladder gave surprisingly high LODs, comparable to those produced by Sanger sequencing. To further explore the LODs of this exon using an additional reference standard, we confirmed that the clinically required variants were detectable at 5% VAF. The challenges faced with ascertaining LODs in this validation study highlight the importance of using multiple types of reference material to gauge LODs on a per exon basis.

The use of the AcroMetrix^™^ Hotspot Frequency Ladder enabled us to assess limits of detection across a broad range of genes and variants in one sample which would otherwise be a costly and time-consuming process. Our assessment of LODs using this reference standard demonstrated inter- and intra-gene variability from the EAFs. This highlights the unsuitability of this platform for the accurate reporting of VAFs and the importance of validating LODs on a wide range of variants if clinical targets span a number of amplicons as LODs may differ by exon [20, 21]. Variation in the observed VAF and EAF was identified, which varied depending upon the gene and exon assessed. In addition, during LOD analysis, we identified observed allelic frequencies with an element of positive or negative bias across repeats; e.g. observed VAFs were consistently higher than expected in some genes and consistently lower than expected in others. We hypothesise that this could be caused by a number of factors including library preparation and sequencing, location of targets within the gene or the surrounding context of the content to be sequence, i.e. large homopolymer regions. The quality of DNA input into the assay may have a large impact on the allelic frequencies observed following sequencing due to the nature of AmpliSeq technology. We are unable to determine the extent of duplicate reads in our final product prior to sequencing using our current protocols and are therefore unable to deduce whether this has impacted the observed ‘bias’. Improvements in DNA quantitation using more sensitive methods as mentioned and assessment of DNA quality by methods such as the ProNex® DNA QC Assay (Promega, NG1002) prior to library preparation would enable control of DNA input quality and to triage samples most applicable to this analytical procedure. In addition, more recent improvements in AmpliSeq panels have resulted in the incorporation of tag-based sequencing in which DNA is barcoded prior to PCR, enabling the identification of duplicate reads. The incorporation of this into the current workflow may negate the current inaccuracies in extrapolating VAFs from this assay.

During the validation process, the need to validate bioinformatic pipelines using multiple software providers became apparent. Despite a large proportion of indels being identified by the IonReporter^™^ routine analysis algorithm, we did identify issues in the routine Ion Reporter^™^ analysis algorithm for the detection of some indels, a type of variant known to present a challenge for NGS analysis [22]. Both false negatives identified in sample sensitivity analysis were indels in *KIT*, which failed to be identified by the routine Ion Reporter^™^ analysis workflow. Adjustment of analysis parameters, namely soft-clipping (the indel was located at the end of amplicon), enabled the successful detection of one indel by the Ion Reporter analysis software. FASTQ files from both indels were further analysed using NextGENe® software (SoftGenetics®) and were successfully identified. Further investigation into indel identification demonstrated that neither Ion Reporter^™^ nor NextGENe® was 100% successful in identification of five indels we ran. Further validation of this bioinformatics workflow, i.e. IonReporter^™^ routine workflow followed by NextGENe® workflow with the same detection parameters on prospective samples, will be required to ensure suitability of the workflow in identification of clinically applicable variants. We believe that this demonstrates the importance of robust and appropriate validation of the bioinformatics pipeline for clinical application and the use of multiple analysis software to ensure detection of all types of variants. We also noted a number of incorrect nomenclature calls on identified variants. We suggest that reporting of NGS-derived results should be made by individuals experienced in the platform, bioinformatics and clinical application of data derived.

In conclusion, with an increasing number of clinically actionable targets requiring a variety of methodologies, an NGS test becomes the more viable option in terms of cost, time and availability of material. For example, within our clinical setting, NSCLC samples now require a plethora of testing across multiple modalities: *ALK* IHC, *ALK* FISH, *ROS1* IHC, *ROS1* FISH, *PDL1* IHC and PCR for *EGFR* and *KRAS* from normally small biopsies with limited material available. NGS enables the assessment of multiple targets using limited input material. The challenge clinical laboratories face is in how much future proofing is appropriate. Here, we have demonstrated that a balance is required between testing current clinically relevant targets and ensuring additional targets which do not currently have clinical utility are a suitable trade-off for sequencing space. It is important to take into account the costly and time-consuming validation/verification process following assay changes when deciding on the size of panel to be implemented into clinical practice.

## Electronic supplementary material

S1 file Reference sample details (XLSX 35 kb)

S2 file Limits of detection (XLSX 23 kb)

S3 file Robustness (XLSX 23 kb)

S4 file Variation (XLSX 42 kb)

## References

[1] Wilkins B ( 2015) The retention and storage of pathological records and specimens, 5th edn

[2] International Organisation for Standardization (2012) ISO 15189:2012 Medical Laboratories -- Requirements for quality and competence 10.1111/j.1423-0410.2002.tb05329.x 12617164

[3] Deans ZC, Costa JL, Cree I, Dequeker E, Edsjo A, Henderson S (2017). Integration of next-generation sequencing in clinical diagnostic molecular pathology laboratories for analysis of solid tumours; an expert opinion on behalf of IQN path ASBL. Virchows Arch.

[4] Thorvaldsdóttir H, Robinson JT, Mesirov JP (2013). Integrative genomics viewer (IGV): high-performance genomics data visualization and exploration. Brief Bioinform.

[5] Robinson JT, Thorvaldsdottir H, Winckler W, Guttman M, Lander ES, Getz G (2011). Integrative genomics viewer. Nat BiotechNOL.

[6] Deans ZC, Watson CC, Charlton R, Ellard S, Wallis Y, Mattocks C, Abbs S (2015) Practice guidelines for targeted next generation sequencing analysis and interpretation

[7] Hall A (2014) Guidelines for diagnostic next generation sequencing. 1–5

[8] Robin JD, Ludlow AT, LaRanger R, Wright WE, Shay JW (2016). Comparison of DNA quantification methods for next generation sequencing. Sci Rep.

[9] Hagemann IS, Devarakonda S, Lockwood CM, Spencer DH (2015) Clinical next-generation sequencing in patients with non – small cell lung cancer. 631–9 10.1002/cncr.29089 25345567

[10] Hadd AG, Houghton J, Choudhary A, Sah S, Chen L, Marko AC (2013). Targeted , high-depth , next-generation sequencing of Cancer genes in formalin-fixed , Paraf fi n-embedded and fine-needle aspiration tumor specimens. J Mol Diagn.

[11] Jennings LJ, Arcila ME, Corless C, Kamel-reid S, Lubin IM, Pfeifer J (2017). Guidelines for validation of next-generation sequencing e based oncology panels a joint consensus recommendation of the Association for Molecular Pathology and College of American pathologists. J Mol Diagn.

[12] Srinivasan M, Sedmak D, Jewell S (2002). Effect of fixatives and tissue processing on the content and integrity of nucleic acids. Am J Pathol.

[13] Do H, Dobrovic A (2015). Sequence artifacts in DNA from formalin-fixed tissues: causes and strategies for minimization. Clin Chem.

[14] Lih C-J, Harrington RD, Sims DJ, Harper KN, Bouk CH, Datta V (2017). Analytical validation of the next-generation sequencing assay for a Nationwide signal-finding clinical trial: molecular analysis for therapy choice clinical trial. J Mol Diagn.

[15] Tumors S, Hovelson DH, Mcdaniel AS, Cani AK, Johnson B, Rhodes K (2015). Development and validation of a scalable next-generation sequencing system for assessing relevant somatic variants in solid tumors. Neoplasia.

[16] Simbolo M, Gottardi M, Corbo V, Fassan M, Mafficini A, Malpeli G (2013). DNA qualification workflow for next generation sequencing of histopathological samples. PLoS One.

[17] Reiman A, Kikuchi H, Scocchia D, Smith P, Tsang YW, Snead D, et al. (2017) Validation of an NGS mutation detection panel for melanoma. 1–7 10.1186/s12885-017-3149-0 PMC5322598 28228113

[18] D’Haene N, Le Mercier M, De Neve N, Blanchard O, Delaunoy M, El Housni H (2015). Clinical validation of targeted next generation sequencing for Colon and Lung cancers. PLoS One.

[19] Greer CE, Peterson SL, Kiviat NB, Manos MM (1991). PCR amplification from paraffin-embedded tissues: effects of fixative and fixation time. Am J Clin Pathol.

[20] Bragg LM, Stone G, Butler MK, Hugenholtz P, Tyson GW (2013). Shining a light on dark sequencing: characterising errors in ion torrent PGM data. PLoS Comput Biol.

[21] Yeo ZX, Wong JCL, Rozen SG, Lee ASG (2014). Evaluation and optimisation of indel detection workflows for ion torrent sequencing of the BRCA1 and BRCA2 genes. BMC Genomics.

[22] Loman NJ, Misra RV, Dallman TJ, Constantinidou C, Gharbia SE, Wain J (2012). Performance comparison of benchtop high-throughput sequencing platforms. Nat Biotechnol.

